# Benralizumab for acute thromboembolism in hypereosinophilic syndrome: a case report

**DOI:** 10.1186/s13223-025-00967-2

**Published:** 2025-05-17

**Authors:** Daiki Nagira, Satoshi Miyamoto, Taishiro Mizukoshi, Atsushi Yanagisawa, Atsushi Funauchi, Kensuke Kanaoka, Hanako Yoshimura, Tatsunori Jo, Masayoshi Higashiguchi, Yujiro Naito, Takayuki Shiroyama, Satoshi Tetsumoto, Haruhiko Hirata, Yoshito Takeda, Atsushi Kumanogoh

**Affiliations:** 1 Department of Respiratory Medicine and Clinical Immunology, Graduate School of Medicine, Osaka University, 2-15, Yamadaoka, Suita, Osaka 565-0871 Japan; 2 Department of Respiratory Medicine and Clinical Immunology, Municipal Hospital, Suita, Osaka, Japan

**Keywords:** Hypereosinophilic syndrome (HES), Thromboembolism, Anticoagulant therapy, Steroid, Interleukin-5 receptor (IL-5 receptor), Benralizumab

## Abstract

**Background:**

Hypereosinophilic syndrome is a group of disorders characterized by organ dysfunction caused by hypereosinophilia, which frequently leads to thromboembolic complications with potentially fatal outcomes. Interleukin-5, a key cytokine that promotes the differentiation and activation of eosinophils, has been identified as a therapeutic target. Anti-interleukin-5 antibody therapy has demonstrated efficacy in reducing eosinophil counts and enabling steroid dose tapering in patients with hypereosinophilic syndrome. This report describes the case of a patient with severe thromboembolism associated with hypereosinophilic syndrome during the acute phase who was successfully treated with benralizumab, an anti-interleukin-5 receptor alpha antibody.

**Case presentation:**

A 22-year-old woman presented with a persistent cough and was diagnosed with eosinophilic pneumonia and portal vein thrombosis. Although eosinophilic pneumonia improved with corticosteroid therapy, thrombotic complications worsened despite additional anticoagulant treatment. The administration of benralizumab led to marked improvement in thrombosis, resulting in clinical recovery.

**Conclusions:**

This case suggests that the early administration of anti-interleukin-5 receptor antibody therapy may be a valuable treatment option for refractory thrombosis.

## Background

Hypereosinophilic syndrome (HES) is a rare and heterogeneous group of disorders characterized by persistent peripheral blood eosinophil counts of ≥ 1500/μL and eosinophil-mediated organ dysfunction. HES is classified into primary, secondary, and idiopathic types [1]. Although rare, its heterogeneous presentation and potential for life-threatening complications pose significant challenges in clinical practice. Thromboembolism, which affects approximately 25% of patients with HES, is one of the most severe complications and contributes to increased mortality [2]. While the molecular mechanisms underlying the contributions of eosinophils to thrombosis remain unclear, one proposed pathway involves integrin-dependent interactions between activated eosinophils and platelets.

Corticosteroids remain the cornerstone of HES treatment, providing rapid control of eosinophilia and organ protection, though long-term steroid dependence and resistance often present significant challenges [3–5]. Recently, anti-IL-5 therapies, such as anti-IL-5 and anti-IL-5 receptor antibodies, have demonstrated efficacy in managing chronic HES by reducing eosinophil counts and steroid-sparing benefits [6]. However, data on their role in acute-phase complications, such as thromboembolism, remain limited.

Here, we report the case of a patient with idiopathic HES with acute-phase massive thromboembolism who was successfully treated with benralizumab.

## Case presentation

**Fig. 1:**
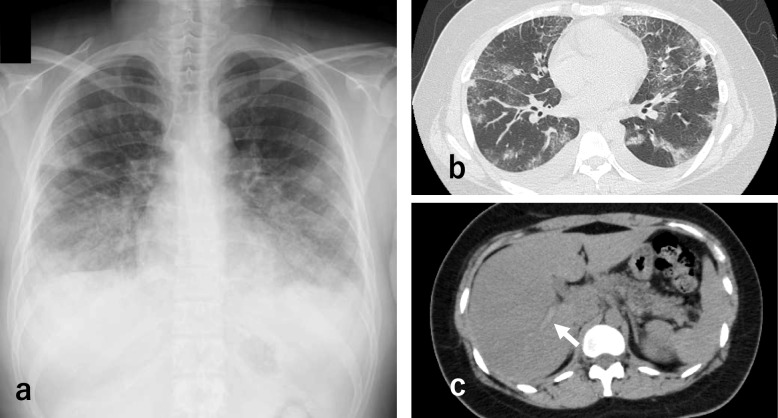
Image findings on admission (day 1). **a** Chest radiograph showing bilateral infiltrative shadows. **b** Chest computed tomography (CT) scan showing bilateral ground-glass opacities and bilateral pleural effusion. **c** Abdominal CT scan showing a portal vein thrombosis

A 22-year-old woman with a history of bronchial asthma was transferred to our hospital because of severe dyspnea and hypereosinophilia. She had no history of smoking, regular medication use, or any relevant family history of thromboembolic disorders. Initially, she was admitted to another hospital with cough, fever, and exertional dyspnea (day 1). Laboratory findings revealed elevated eosinophil counts and inflammatory marker levels. Chest computed tomography (CT) demonstrated bilateral ground-glass opacities and portal vein thrombosis (Fig. 1). Despite antibiotic therapy, her condition deteriorated. Bronchoscopy performed on day 4 revealed a marked eosinophilic predominance in bronchoalveolar lavage fluid. Differential cell counts showed eosinophils at 86% (reference range: < 1%), neutrophils at 5% (reference range: < 3%), lymphocytes at 6% (reference range: 10–15%), and macrophages at 3% (reference range: ≥ 85%). The total cell count was 5.77 × 10⁶ cells/mL (reference range: 0.7–2 × 10^5^ cells/mL). Although methylprednisolone pulse therapy was initiated for the suspected eosinophilic pneumonia, her respiratory status worsened, necessitating intubation and transfer to our hospital on day 5.

At the time of referral, her vital signs were as follows: body temperature, 37.1 °C; blood pressure, 125/73 mmHg; pulse, 52 beats/min (regular); respiratory rate, 22 breaths/min; and oxygen saturation, 98% (mechanical ventilation in A/C mode; fraction of inspired oxygen, 0.6; pressure support, 12 cm H_2_O; positive end-expiratory pressure, 10 cmH_2_O). Physical examination revealed coarse breath sounds bilaterally, without wheezing and erythematous lesions on both lower extremities.

Laboratory examinations revealed the following results: white blood cell count, 124,000/µL (reference range: 3,300–9,400/µL); eosinophils, 10.6% (reference range: 0.0–7.0%); platelet count, 64,000/µL (reference range: 13,000–32,000/µL); C-reactive protein, 5.5 mg/dL (reference range: 0.0–0.2 mg/dL); and fibrin degradation products, 91 µg/mL (reference range: 0.0–5.0 µg/mL). Skin biopsy revealed eosinophilic infiltration in and around the blood vessels in the dermis and subcutaneous tissue. Serum IgE level was markedly elevated at 3,630 IU/mL (reference range: 0.0–173 IU/mL). Tests for the *FIP1L1/PDGFRA* fusion gene, antinuclear antibodies, myeloperoxidase anti-neutrophil cytoplasmic antibodies, proteinase 3 anti-neutrophil cytoplasmic antibodies, β-d-glucan, and cryptococcal antigen yielded negative results. Angiotensin-converting enzyme and IgG levels were within normal ranges. Serologic tests for parasitic infections were performed; all results were negative. With no evidence of secondary or neoplastic HES, a diagnosis of idiopathic HES was established.

**Fig. 2:**
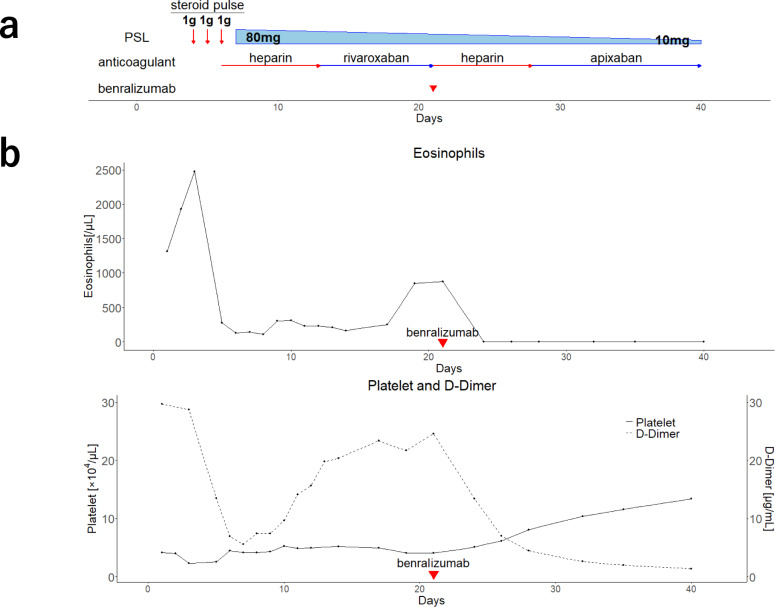
Treatment course and blood parameter trends. **a** Schematic treatment summary. **b** Peripheral blood eosinophil counts, D-dimer levels, and platelet counts during the clinical course

**Fig. 3:**
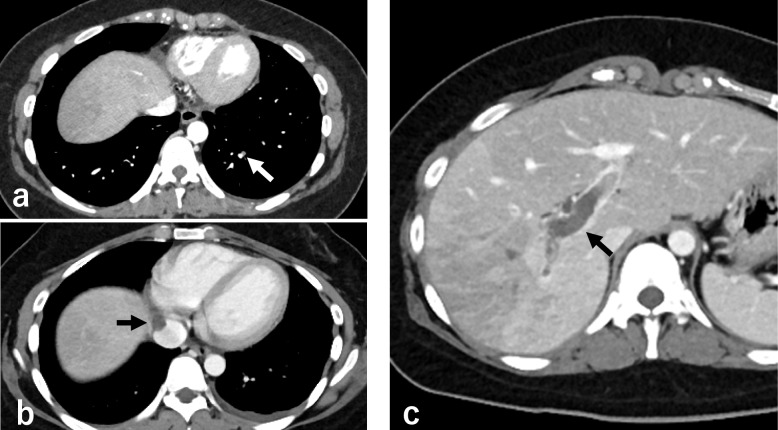
Abdominal contrast CT image findings on day 20. **a** Pulmonary embolism. **b** Inferior vena cava thrombosis. **c** Portal vein thrombosis

**Fig. 4:**
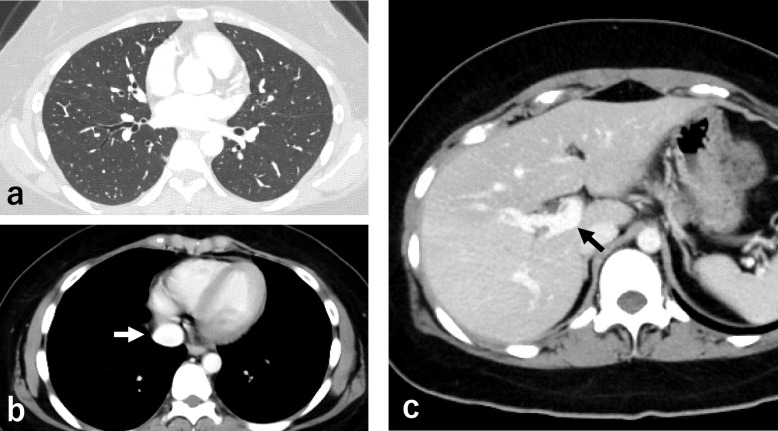
Chest and abdominal CT image findings after improvement. **a** CT scan on day 20 showing improvement in pulmonary infiltrates. **b**, **c** Contrast CT scan on day 40 showing a significant reduction in the size of multiple thrombi

The clinical course of the patient is shown in Fig. 2. Methylprednisolone pulse therapy and continuous intravenous infusion of heparin (22,000 units/day) were administered for 3 days. As the initial administration of heparin was complicated by endobronchial bleeding, the heparin dose was subsequently adjusted to maintain the activated partial thromboplastin time (aPTT) at approximately 37 s, corresponding to approximately 1.5 times the pre-heparin baseline, to minimize the risk of further bleeding. Following respiratory improvement and decreased eosinophil counts (230 cells/µL), mechanical ventilation was successfully discontinued on day 11. However, despite the ongoing steroid treatment and continuous oral rivaroxaban, eosinophil levels rose again (844 cells/µL), and D-dimer levels remained elevated (21.6 µg/mL, reference range: 0.0–1.0 µg/mL). Therefore, contrast-enhanced CT was performed on day 20, revealing that inferior vena cava thrombosis and pulmonary embolism had developed in addition to persisting portal vein thrombosis (Fig. 3), despite the improvement in pulmonary infiltrates (Fig. 4a). Given the progression of thrombosis and suspected involvement of eosinophils in thrombus formation, rapid elimination of eosinophils was deemed necessary. Therefore, the continuous intravenous infusion of heparin (22,000 units/day) was resumed, and benralizumab was administered on day 21 to target eosinophil-mediated thrombosis. However, because the aPTT was excessively prolonged to 80 s, the heparin dose was reduced to 15,000 units/day; thereafter, the aPTT was maintained at approximately 50 s. Subsequently, the blood eosinophil count decreased to 0 cells/µL, with a concurrent reduction in D-dimer levels (2.6 µg/mL). Abdominal ultrasonography on day 31 demonstrated resolution of portal vein thrombosis, and follow-up contrast-enhanced CT on day 40 revealed significant reduction in the size of multiple thrombi (Fig. 4b, c). Treatment was switched from heparin to oral apixaban, and the patient was discharged on day 42. The follow up over 6 months with gradual dose tapering of steroids and continuation of apixaban administration showed no recurrence.

## Discussion and conclusions

This case report suggests that anti-IL-5 receptor antibody therapy may be effective for HES-related thromboembolism resistant to anticoagulant therapy. HES is known to cause severe thromboembolism, which significantly affects patient prognosis. Previous studies have reported that approximately 25% of patients with HES develop thrombosis, and thrombotic events following diagnosis are strongly associated with poor outcomes and increased mortality [2]. Among eosinophilia-related thromboembolic conditions, pulmonary thromboembolism is the most common manifestation (52%), with HES being the most frequent underlying cause (30%) [7]. Despite the clinical significance of HES-related thromboembolism, no established guidelines exist for its prevention and treatment. Although the efficacy of anticoagulant therapy remains controversial, it is recommended for high-risk patients with conditions such as intracardiac thrombosis, deep vein thrombosis, and recurrent thromboembolism using agents such as warfarin, heparin, low-molecular-weight heparin, or direct oral anticoagulants [8–11]. However, in cases complicated by thrombosis, consumptive thrombocytopenia may occur, limiting anticoagulation owing to bleeding tendencies. Treatment discontinuation may be considered when eosinophilia is controlled and no residual thrombi are present [8, 9]. Although previous reports have shown successful treatment of HES-associated deep vein thrombosis using corticosteroids combined with direct oral anticoagulants [4], no standard treatment exists for thromboembolism refractory to anticoagulant therapy.

Recently, IL-5-targeted therapies have emerged as promising treatment options for HES. Mepolizumab, an anti-IL-5 antibody, inhibits the binding of IL-5 to its receptor on eosinophils and has been approved by the Food and Drug Administration for the treatment of idiopathic HES in patients aged ≥ 12 years [12, 13]. It has also demonstrated efficacy in treating eosinophilic vasculitis, a complication of HES [14]. In contrast, benralizumab is a monoclonal antibody that directly binds to IL-5 receptor alpha and exerts a rapid and specific eosinophil-depleting effect through antibody-dependent cell-mediated cytotoxicity [15].

In the present case, eosinophils were considered to be directly involved in thrombus formation, leading to the use of benralizumab because of its potential to rapidly eliminate eosinophils. While benralizumab has shown efficacy in reducing eosinophil counts and facilitating steroid dose tapering in patients with HES [16], its thrombus-suppressing effects in acute-phase HES-related thromboembolism have not been previously reported. Clinical evidence supports the involvement of eosinophils in thrombus formation; however, the precise molecular mechanisms remain unclear. One proposed mechanism involves CCL11, an eosinophil-specific chemotactic factor that activates eosinophils and induces von Willebrand factor expression on the vascular endothelium, thereby promoting platelet adhesion. This process facilitates thrombus formation and eosinophil aggregation through integrin-dependent interactions with the platelets. These interactions trigger eosinophil extracellular traps, which, modified by eosinophil granules, activate platelets and propagate thrombus formation [17]. Additionally, thrombin-activated platelets can stimulate eosinophils to secrete IL-5, potentially enhancing eosinophil extracellular trap formation. Previous studies have reported that low blood eosinophil counts are also predictive of increased risk of thrombosis, suggesting that eosinophils are consumed during the thrombus formation process [18]. Thus, anti-IL-5 receptor antibody therapy has been suggested as a potential therapeutic strategy for eosinophil-associated thrombosis by directly eliminating eosinophils at thrombotic sites, thereby effectively disrupting the pathological cycle.

This case demonstrates the successful resolution of a hypercoagulable state through anti-IL-5 receptor antibody therapy in a patient with acute-phase HES refractory to conventional corticosteroid and anticoagulant therapies. Despite the failure of these conventional treatments to achieve thrombolysis, the addition of anti-IL-5 receptor antibody therapy reduced eosinophil counts, enhanced the efficacy of anticoagulant therapy, and increased platelet counts. This represents a rare instance in which anti-IL-5 receptor antibody therapy was used as remission induction therapy for severe thrombosis in a patient with HES. These findings suggest that the early administration of anti-IL-5 receptor antibody therapy may be a valuable treatment option for refractory thrombosis. As similar cases have not been reported, further case accumulation is essential to establish the efficacy and role of anti-IL-5 receptor antibody therapy in clinical practice.

## Data Availability

No datasets were generated or analysed during the current study.

## Abbreviations

CT: Computed tomography
HES: Hypereosinophilic syndrome
IL: Interleukin
